# Postfire soil water repellency in piñon–juniper woodlands: Extent, severity, and thickness relative to ecological site characteristics and climate

**DOI:** 10.1002/ece3.3039

**Published:** 2017-05-22

**Authors:** Daniel L. Zvirzdin, Bruce A. Roundy, Nicholas S. Barney, Steven L. Petersen, Val J. Anderson, Matthew D. Madsen

**Affiliations:** ^1^ Bureau of Land ManagementElkoNVUSA; ^2^ Department of Plant and Wildlife SciencesBrigham Young UniversityProvoUTUSA

**Keywords:** climate change, ecological site characteristics, hydrophobicity, postfire restoration, soil organic matter, soil water repellency, woodland encroachment

## Abstract

Wildfires can create or intensify water repellency in soil, limiting the soil's capacity to wet and retain water. The objective of this research was to quantify soil water repellency characteristics within burned piñon–juniper woodlands and relate this information to ecological site characteristics. We sampled soil water repellency across forty‐one 1,000 m^2^ study plots within three major wildfires that burned in piñon–juniper woodlands. Water repellency was found to be extensive—present at 37% of the total points sampled—and strongly related to piñon–juniper canopy cover. Models developed for predicting SWR extent and severity had *R* ^2^_adj_ values of 0.67 and 0.61, respectively; both models included piñon–juniper canopy cover and relative humidity the month before the fire as coefficient terms. These results are important as they suggest that postfire water repellency will increase in the coming years as infilling processes enhance piñon–juniper canopy cover. Furthermore, reductions in relative humidity brought about by a changing climate have the potential to link additively with infilling processes to increase the frequency and intensity of wildfires and produce stronger water repellency over a greater spatial extent. In working through these challenges, land managers can apply the predictive models developed in this study to prioritize fuel control and postfire restoration treatments.

## 1 INTRODUCTION

Since the late 19th century, global temperature has increased by 0.85°C (IPCC, 2013), models predict even more abrupt temperature changes occurring by the end of the 21st century (Fischer & Schär, 2010; Ganguly et al., 2009; IPCC, 2013). If these predictions prove accurate, different strategies and techniques may be needed to effectively manage soil resources (Orwin et al., 2015). One soil property that may be increasingly important as global climate change progresses is soil water repellency (SWR) (Goebel, Bachmann, Reichstein, Janssens, & Guggenberger, 2011; Shakesby & Doerr, 2006). This soil condition develops as hydrophobic molecules released by plant tissues and microbes coat soil particles (Doerr, Shakesby, & Walsh, 2000; McGhie & Posner, 1981), creating a nonpolar soil layer (DeBano, Savage, & Hamilton, 1976; Letey, 2001). The impact of SWR is found in both natural and anthropogenic soils (DeBano, 1981; Doerr et al., 2000; Wallis & Horne, 1992) and is predicted to increase worldwide with the further development of climate change (Goebel et al., 2011).

The highly positive water entry pressure of water‐repellent soil degrades infiltration and percolation rates (DeBano, 1971; Doerr & Thomas, 2000; Doerr et al., 2003; Madsen, Zvirzdin, Petersen, et al., 2011; Pierson, Robichaud, & Spaeth, 2001). This initiates a cascade of ancillary effects including reduced soil moisture (Madsen, Zvirzdin, Petersen, et al., 2011; Wallis & Horne, 1992), enhanced runoff and erosion (Benavides‐Solorio & MacDonald, 2001; DeBano, 2000; Leighton‐Boyce, Doerr, Shakesby, & Walsh, 2007; Martin & Moody, 2001), and reduced postfire restoration success (Letey, 2001; Madsen et al., 2012; Wallis & Horne, 1992). In turn, these direct effects can indirectly decrease resistance to weed invasion and accelerate site degradation (Young & Evans, 1978).

Fire triggers a range of responses in the soil relative to SWR. These responses range from the dissipation of SWR in areas where it was present prior to fire, to significant increases in the attributes of the water‐repellent layer (Doerr, Shakesby, Dekker, & Ritsema, 2006; Doerr, Woods, Martin, & Casimiro, 2009; Jiménez‐Pinilla, Doerr, et al., 2016; Jordan, Zavala, Mataix‐Solera, Nava, & Alanis, 2011; Pierson et al., 2008; Zavala, Granged, Jordán, & Bárcenas‐Moreno, 2010). Within fires that generate extreme temperatures, water repellency is often destroyed at the soil surface and intensified slightly below (DeBano, 1971; Doerr, Shakesby, & MacDonald, 2010; Doerr et al., 2006; Robichaud & Hungerford, 2000). Previous to fire, soil features such as surface roughness, vegetation, litter, and soil organic matter mitigate the effects of SWR (DeBano, 2000; Leighton‐Boyce et al., 2007). As most of these balancing variables are removed with fire (Doerr et al., 2010; Shakesby, Coelho, Ferreira, Terry, & Walsh, 1993), in many cases, the effects of SWR are likely most fully realized postfire.

In the western United States, the magnitude of SWR effects has been found to be related to the extent and strength of the water‐repellent layer (Doerr et al., 2009; Pierson et al., 2001; Woods, Birkas, & Ahl, 2007). Typically, overland flow generated in water‐repellent zones infiltrates as it contacts adjacent hydrophilic patches or conduits (Shakesby, Doerr, & Walsh, 2000). When SWR continuity is high, hydrophilic patches are sparse and may be inadequate to accommodate surface runoff (Woods et al., 2007). Woods et al. (2007) intensively studied the continuity of SWR at multiple spatial scales and concluded that whenever water‐repellent soils comprise more than 75% of sampled points (i.e., 75% SWR extent) within a slope or watershed, there is a high probability that continuous overland flow will be generated.

Piñon (*Pinus* L.) and juniper (*Juniperus* L.) woodlands have replaced historically dominant sagebrush/bunchgrass vegetation types throughout the Intermountain West (Miller & Rose, 1995; Miller, Tausch, Macarthur, Johnson, & Sanderson, 2008). These woodlands now occupy over 40 million hectares (Romme et al., 2009). In replacing historic plant communities, piñon–juniper woodlands have shifted fuel conditions from primarily light understory fuels to heavier canopy fuels prone to intense stand‐replacing crown fires (Gruell, 1999; Miller & Tausch, 2001).

Postfire recovery of arid ecosystems in the Intermountain West is often poor (Arkle et al., 2014; Knutson et al., 2014; Pyke, 2011). In piñon–juniper woodlands, recovery is dependent on the extent that physical and biological processes have been altered (Briske, Fuhlendorf, & Smeins, 2005; Miller & Tausch, 2001). If extensive, alterations can trigger feedback mechanisms that lead to the crossing of ecological thresholds (Briske, Bestelmeyer, Stringham, & Schaver, 2008; Davenport, Breshears, Wilcox, & Allen, 1998). When thresholds are crossed in piñon–juniper woodlands, sites transition to undesirable alternative stable states, recovery from which may be difficult or impossible (Miller, Svejcar, & Rose, 2000; Pyke, 2011).

Soil water repellency has been documented both pre‐ and postfire in piñon–juniper woodlands (Jaramillo, Dekker, Ritsema, & Hendrickx, 2000; Madsen, Chandler, & Belnap, 2008; Madsen, Zvirzdin, Petersen, et al., 2011, 2012; Rau, Chambers, Blank, & Miller, 2005; Roundy, Blackburn, & Eckert, 1978; Scholl, 1971; Williams et al., 2016). However, studies have been localized and the continuity and strength of this soil condition across a range of ecological sites have not been clearly shown. In addition, links between SWR and specific ecological site characteristics have not been established; land managers have no ready substitute for in situ data when seeking to quickly identify areas where SWR may be a problem.

Links between SWR and specific site characteristics have been established in many other ecosystems (Doerr et al., 2000 and references therein). Of the many tested characteristics, soil organic matter content (Atanassova & Doerr, 2010; Mataix‐Solera et al., 2007; Scholl, 1971; Varela, Benito, & de Blas, 2005), pH (Hurraß & Schaumann, 2006; Martínez‐Zavala & Jordán‐López, 2009; Mataix‐Solera et al., 2007; Steenhuis et al., 2001), texture (DeBano, 1991; Jordan, Zavala, Nava, & Alanis, 2009; Mataix‐Solera et al., 2007), soil moisture (Doerr et al., 2000; Letey, 2001), burn severity (Jordan et al., 2011; Pierson, Carlson, & Spaeth, 2002; ), litter (McGhie & Posner, 1981), vegetation type/land use (Doerr et al., 2000, 2006; Jiménez‐Pinilla, Lozano, et al., 2016; Mataix‐Solera et al., 2007; Tessler, Wittenberg, Malkinson, & Greenbaum, 2008), and topography (Doerr et al., 2009; Pierson et al., 2002; Tessler et al., 2008) have received the most attention. While consistent relationships between these variables and SWR have been found in some studies (Jordán, Zavala, Mataix‐Solera, & Doerr, 2013), inconsistencies between studies are common (Doerr et al., 2000, 2006; Jiménez‐Morillo et al., 2016; Martínez‐Zavala & Jordán‐López, 2009). This inconsistency precludes the extrapolation of documented links between site characteristics and SWR to other systems where SWR data are lacking.

The objectives of this research were to: (1) quantify the extent and severity of SWR, and the thickness of the water‐repellent layer within burned piñon–juniper woodlands across a range of ecological sites, (2) determine which ecological site characteristics are most closely related to SWR within these woodlands, and (3) develop predictive models of SWR that could be used by land managers without the need to gather extensive in situ data. It was hypothesized that SWR would have a close association with piñon and juniper trees and that soil attributes and topography would play important roles in defining SWR extent and severity and thickness of the water‐repellent layer.

## 2 METHODS

### 2.1 Site selection

Three major wildfires that burned in the state of Utah in 2009 were selected for SWR sampling: Big Pole, Broken Ridge, and Mill Flat. These fires were ignited on 25 July, 2 August, and 7 August, respectively, and burned 17,775, 1,995, and 4,856 ha, respectively. The Big Pole fire is located 10 miles west of Grantsville (40°35ʹN 112°40ʹW), and the Broken Ridge and Mill Flat fires are found 40 miles northwest and 20 miles southwest of Cedar City, respectively (38°06ʹN 113°36ʹW and 37°30ʹN 113°20ʹW). To increase the likelihood of capturing the natural variability typical in SWR, study sites were selected based on five ecological site characteristics shown important to the formation of SWR in other systems: soil texture, soil pH, soil organic matter content, precipitation, and heat load. Precipitation and heat load were used as proxies for soil moisture and topography. Heat load is an index of potential soil heating resultant from the timing of solar radiation relative to aspect, slope, and latitude (McCune & Keon, 2002). In addition to their support in the literature, these variables were selected for their public availability in GIS format, which enabled us to remotely identify suitable study plots.

Geospatial fire boundary data for the three fires were postprocessed to represent piñon–juniper woodlands exclusively. Soil and precipitation data from within the fire boundary were obtained from the NRCS Soil Data Mart (www.soildatamart.nrcs.usda.gov) and the PRISM Climate Group (www.prism.oregonstate.edu). Heat load data were developed using methods established by McCune and Keon (2002), using data derived from 10 m digital elevation models (DEMs), acquired from the Utah Automated Geographic Reference Center (www.gis.utah.gov).

To distribute study sites across the five ecological site characteristics, a shapefile of each characteristic was obtained and broken into three equal interval categories (i.e., low, medium, and high) in ArcGIS 9.3 (ESRI Corp, Redlands, CA, USA). All factorial combinations of the three categories by the five ecological site characteristics were identified. Polygons representing each of these unique combinations—a total of 41—were created, and a random point was generated within. These random points became the southwest corners of the study plots.

### 2.2 Sampling protocol

Random points were located in the field using a handheld Trimble GeoXH global positioning system (GPS) receiver (Trimble, Sunnyvale, CA, USA). At each random point, a 30 × 33 m (~1,000 m^2^) plot was established. The 33 m axis was oriented N–S. Five, 24 m transects were systematically placed along the 30 m E‐W axis at 2, 7, 15, 23, and 28 m. Measurements were taken every 3 m along each 24 m transect for a total of nine sampling points per transect and 45 sampling points per plot. Variability in soil moisture was indirectly controlled for by sampling during the dry season (June–August), and delaying the sampling of sites for at least 1 week, following measurable precipitation events, and sampling sites indiscriminately.

Water repellency was measured at each sampling point with the water drop penetration time (WDPT) test (Krammes & DeBano, 1965). Soils were considered water repellent if WDPT time exceeded five seconds (Bisdom, Dekker, & Shoute, 1993). Where SWR was found, thickness of the water‐repellent layer was determined by performing WDPT tests every 5 mm. For sampling points that had field WDPTs over 2 min, a soil sample was collected and WDPT tests were conducted in the laboratory.

Following SWR sampling, the nearest woody plant to each point was located. Species was determined, distance between the sampling point and the trunk and canopy edge (i.e., the furthest horizontal projection) of that species was measured, and microsite (i.e., tree mound or interspace) was recorded. If the nearest woody species was a piñon or juniper tree, height and width, trunk diameter, and burn severity were measured. Tree crown width of piñon and juniper trees was defined as the average of the overall widest diameter and the widest diameter perpendicular to this first diameter. Trunk diameter was measured just above the root crown. Burn severity was determined based on a subjective five point scale: (1) burned piñon–juniper trees with the majority of the needles still attached, (2) needles lacking, major branches still present, (3) major branches lacking, trunk still intact, (4) trunk hollowed out or otherwise not intact, but still present, and (5) trunk largely lacking.

At the plot level, ten trees were randomly selected for radial growth core extraction. Radial growth cores were taken 30 cm above the soil surface using an increment bore (Haglöf Company Group, Långsele, Sweden). To accurately determine the age of piñon–juniper individuals, cross‐dating is necessary (Despain, 1989). This level of accuracy was outside the scope of this project; consequently, the absolute age of the cored trees was not determined.

Eight random soil subsamples were taken. Four from interspaces and four from tree/shrub mound zones. The top 4 cm of mineral soil was taken as this is typically where water‐repellent organic residues concentrate within the profile (Madsen, Zvirzdin, Petersen, et al., 2011). Subsamples were combined for each zone. Acidity, soil organic matter, and texture were analyzed in the laboratory using saturation extract (Rhodes, 1982), dichromate oxidation (Walkley & Black, 1934), and hydrometer (Day, 1965) methods.

Heat load data were extracted at the plot level from the DEM dataset retrieved previously in the study site selection process, as were elevation, aspect, and slope. Piñon–juniper canopy cover was manually digitized from 1‐m resolution digital orthophoto quarter quads (DOQQ) acquired from the National Agriculture Imagery Program (US Department of Agriculture, 2010). Climate data, including annual and July 2009 (the month before ignition for all three fires in this study) precipitation, minimum and maximum temperature, and dewpoint, were extracted for each study site from the Prism Climate Group dataset (PRISM Climate Group, 2010). Relative humidity was calculated from temperature and dewpoint using the Goff‐Gratch Equation (Ahrens, 2009).

### 2.3 Data analysis

Statistical analyses were conducted at both the sampling point and plot levels using JMP 10.8 (SAS Institute; Cary, NC, USA). Soil water repellency severity data were classified by WDPT results, according to Bisdom et al. (1993): slight (5–60 s), strong (60–600 s), severe (600–3,600 s), and extreme (>3,600 s). In the plot level analysis, the thickness of the water‐repellent layer and the severity of SWR were averaged across all water‐repellent sampling points within a plot. The normality of continuous data was tested in normal quantile plots. Data not following a normal distribution were log transformed as appropriate. For the general linear models developed, equal variance and independence were tested with Levene's Equal Variance test and the Durbin–Watson test.

In the sampling point analysis, response variables included SWR presence, severity, and the thickness of the water‐repellent layer; explanatory variables included microsite, distance to the trunk and canopy (i.e., the furthest horizontal projections) of the nearest woody species, and woody species composition. Data from all 1,845 sampling points (41 sites × 45 sampling points per site = 1,845) were pooled in the sampling point analysis, and site was set as a random effect. Mantel's test was performed in R (R Core Development Team; Vienna, Austria) to verify that these data were not spatially autocorrelated (Sokal & Rohlf, 1995). A significance level of *p* < .05 was used for all comparisons. As in the plot level analysis, normality was tested and data were transformed as appropriate.

Comparisons of SWR extent between microsites (i.e., canopy and intercanopy regions) and between species were conducted with Fisher's exact test. Comparisons of SWR severity and the thickness of the water‐repellent layer between microsites were conducted with Welch's test. Differences in the distance to the canopy and distance to the trunk between sampling points where SWR was present/absent were also determined with Welch's test. Welch's test was used due to the non‐normal distribution and unequal variance of these response data (Skovlund & Fenstad, 2001).

In the plot level analysis, SWR extent was defined as the percentage of points within a plot where water‐repellent conditions were observed. Response variables included SWR extent and severity and the thickness of the water‐repellent layer. The plot level explanatory variable dataset was refined prior to analysis to eliminate correlated variables. The final explanatory dataset included piñon–juniper canopy cover, height, tree ring count, trunk diameter, and burn severity, tree mound soil pH and clay content, annual minimum and maximum temperature, and July 2009 relative humidity.

A model index was developed for each SWR characteristic from this refined plot level dataset. In the model selection process, (1) models were ranked based on their Bayesian Information Criterion (BIC) values; that is, models with the lowest BIC values ranked highest (Burnham & Anderson, 1998), (2) all coefficient estimates were required to be significant, and (3) models were limited to three coefficients due to sample size. Outliers and influential points were identified using studentized residuals and Cook's distance values (Cook & Weisburg, 1982).

From within the final model list for each SWR characteristic, a single predictive model was selected. These models were selected based on their parsimony and the remote accessibility of their coefficient terms. Model parsimony was a key criterion in the selection process as it reduces the likelihood that the selected model is an artifact of the data, rather than the observed phenomenon. Remote accessibility ensures that selected models can be used in the absence of in situ data.

The relative importance of individual ecological site characteristics was determined through the following procedure. A model average with a three coefficient maximum was developed for each SWR characteristic, and a model averaged formula was derived. Within each formula, median field values were input for all coefficients in the model average but one. For this one coefficient, coefficient *x*, the maximum field value was input and the product of the formula, Max_*x*_, was recorded. This process was repeated using the minimum field value for coefficient *x* to produce Min_*x*_. Max_*x*_ and Min_*x*_ were then computed for all coefficients. A normalized influence statistic, Norm_*x,*_ was calculated from these values for each ecological site characteristic according to equation (1):

(1) $${\text{Norm}}_{x}\;=\;\frac{{\text{Max}}_{x}\;-\;{\text{Min}}_{x}}{\sum_{x=1}^{n}\left({\text{Max}}_{x}\;-\;{\text{Min}}_{x}\right)}$$

where the difference between Max_*x*_ and Min_*x*_ for a single coefficient is divided by the summed difference between Max_*x*_ and Min_*x*_ for all coefficients. Norm_*x*_ is a measure of how much the model average changes when just one coefficient is varied from its maximum to minimum as compared to when all coefficients are varied from their maximums to minimums. It asks, of all the variability possible within the model average, how much is due to variation in coefficient *x*? Norm_*x*_ values range between 0 and 1; more influential coefficients have higher Norm_*x*_ values.

## 3 RESULTS

### 3.1 Soil water repellency extent and severity, and the thickness of the water repellent layer

Across the study, SWR was found at 37% of all points tested. SWR extent exceeded 75% within 10% of the study plots. Among fires, 0%, 10%, and 38% of sites at Big Pole, Broken Ridge, and Mill Flat exceeded 75% SWR extent within a study plot. In the tree/shrub mound zones of woody species, 71% of sampling points were water repellent and 16% of interspace points were water repellent (*p* < .001). Between microsites, SWR severity (i.e., WDPT) and the thickness of the water‐repellent layer were significantly greater for tree/shrub mounds as compared to interspaces, averaging 1,476 s and 858 s for WDPT (*p* = .008), and 1.90 cm and 1.42 cm for the thickness of the water‐repellent layer (*p* = .003). In the interspaces, water‐repellent points were found closer to the canopy edges of woody species (0.92 m) on average as compared to nonwater repellent points (1.98 m) (*p* < .001).

Soil water repellency extent and severity, and the thickness of the water‐repellent layer were higher below the canopy of piñon as compared to Utah juniper. Between the two species, SWR extent averaged 79% and 69% (*p* = .017), WDPTs averaged 2,328 s and 1,188 s (*p* = .020), and the thickness of the water‐repellent layer averaged 2.86 cm and 1.62 cm (*p* < .001).

Among all woody species, the percentage of tree/shrub mound sample points that were water repellent varied: Utah juniper (69%), singleleaf piñon (79%), two needle piñon (*Pinus edulis* Engelm., 92%), Gambel oak (*Quercus gambelii* Nutt., 60%), Stansbury cliffrose (*Purshia stansburiana* (Torr.) Henrickson, 73%), and Saskatoon serviceberry (*Amelanchier alnifolia* (Nutt.) Nutt. ex M. Roem., 70%). Soil water repellency was also found under big sagebrush (*Artemisia tridentata* Nutt.), antelope bitterbrush (*Purshia tridentata* (Pursh) DC.), Sonoran scrub oak (*Quercus turbinella* Greene), alderleaf mountain mahogany (*Cercocarpus montanus* Raf.), curl‐leaf mountain mahogany (*Cercocarpus ledifolius* Nutt.), Yucca (Yucca L.), and pointleaf manzanita (*Arctostaphylos pungens* Kunth), but sample sizes were too small (i.e., <10) for us to be confident in reporting summary statistics.

### 3.2 Ecological site characteristic modeling

Models of SWR extent were proficient in predicting variance in the dependent variable; *R* ^2^_adj_ values in the top ten models ranged from 0.64 to 0.76. The top model included the following three coefficients: piñon–juniper canopy cover, tree mound soil pH, and average tree ring count (Table 1). Models of SWR severity showed relatively weaker correlations; *R* ^2^_adj_ values within the top ten models ranged from 0.45 to 0.61. The top model included the following two coefficients: piñon–juniper canopy cover and average relative humidity for July 2009 (Table 1). Models of the thickness of the water‐repellent layer were weak, only six models met the established criterion; the top model produced a 0.44 *R* ^2^_adj_ and included piñon–juniper canopy cover and tree mound clay content.

**Table 1:** Top models of soil water repellency (SWR) extent and severity, and the thickness of the water‐repellent layer in piñon–juniper (PJ) woodlands

Model #	Explanatory variables^a^	*K* b	*R* ^2^_adj_ c	BIC^d^	*w*_*i*_ e
Extent					
1	PJ canopy cover, Tree mound soil pH, PJ tree ring count	3	0.76	−46.7	0.59
2	PJ canopy cover, Tree mound soil pH, July 2009 relative humidity	3	0.74	−44.7	0.21
3	PJ canopy cover, Tree mound clay, July 2009 relative humidity	3	0.73	−42.6	0.08
4	PJ canopy cover, Tree mound clay, PJ tree ring count	3	0.73	−42.5	0.07
5	PJ canopy cover, Tree mound clay, Annual min. temperature	3	0.71	−40.2	0.02
6	PJ canopy cover, Tree mound clay	2	0.68	−38.7	0.01
7	PJ height, Tree mound soil pH, PJ tree ring count	3	0.69	−38.2	0.01
**8**	**PJ canopy cover, July 2009 relative humidity**	**2**	**0.67**	−**38.0**	**0.01**
9	PJ canopy cover, PJ tree ring count	2	0.67	−35.3	0.00
10	PJ canopy cover, Annual min. temperature	2	0.64	−34.3	0.00
Severity					
**1**	**PJ canopy cover, July 2009 relative humidity**	**2**	**0.61**	**39.2**	**0.90**
2	Tree mound soil pH, July 2009 relative humidity, PJ trunk diameter	3	0.58	44.2	0.04
3	July 2009 relative humidity, Annual min. temperature, PJ trunk diameter	3	0.57	44.7	0.02
4	Annual max. temperature, Annual min. temperature, PJ trunk diameter	3	0.56	45.1	0.01
5	July 2009 relative humidity, Annual min. temperature	2	0.53	45.7	0.01
6	PJ canopy cover, Annual max. temperature, Annual min. temperature	3	0.56	46.3	0.01
7	Annual min. temperature	1	0.45	49.4	0.00
8	Tree mound soil pH, July 2009 relative humidity	2	0.48	49.6	0.00
9	Tree mound soil pH, PJ tree ring count, PJ trunk diameter	3	0.50	50.6	0.00
10	Tree mound soil pH, Annual max. temperature, PJ trunk diameter	3	0.48	52.0	0.00
Thickness					
1	Tree mound clay, PJ canopy cover	2	0.44	80.9	0.88
2	Tree mound clay	1	0.31	85.0	0.11
3	PJ canopy cover	1	0.20	91.8	0.00
4	Tree mound soil pH	1	0.16	93.5	0.00
5	PJ height	1	0.12	95.2	0.00
6	Annual average min. temperature	1	0.11	95.9	0.00

Models endorsed for the prediction of SWR in bold.

a Variables included in the model.

b Number of model terms.

c Adjusted coefficient of determination.

d Baysian information criterion value.

e Model weight.

As per the criteria outlined in the methods, the eighth model of SWR extent and the first model of SWR severity were selected for use in predicting SWR. Both models included piñon–juniper canopy cover and relative humidity as coefficients, the estimates of which are provided in Table 2. Top models that included tree mound soil clay content, soil pH, and soil organic matter were not considered for predicting SWR. These variables are poorly related to soil clay content, soil pH, and soil organic matter data in the NRCS dataset (*r* = .11, .30, and .30) and therefore fail to meet our criteria. A predictive model of the thickness of the water‐repellent layer was not endorsed, all models failed to meet the established criteria.

**Table 2:** Estimates and *p*‐values of coefficients in the endorsed predictive models of soil water repellency extent and severity

Coefficient	Estimate	*p*‐value
Extent		
Intercept	0.288	.022
Piñon–juniper canopy cover	0.019	<.001
July 2009 relative humidity	−0.013	.005
Severity		
Intercept	3.178	<.001
Piñon–juniper canopy cover	0.034	<.001
July 2009 relative humidity	−0.079	<.001

Within the model, average SWR extent, and piñon–juniper canopy cover had the strongest influence (Norm_*x*_ = .50) (Table 3). Tree mound soil pH and average tree ring count ranked second and second in influence, having Norm_*x*_ values of .25 and .17. All of the remaining variables played minor roles. The two most important variables for SWR severity were piñon–juniper canopy cover and July 2009 relative humidity (Norm_*x*_ = .50 and .36). The two most important variables for the thickness of the water‐repellent layer were tree mound clay content and piñon–juniper canopy cover (Norm_*x*_ = .48 and .39).

**Table 3:** Relative influence of ecological site characteristics on soil water repellency, ranked based on their ability to induce variation within a model average having a three maximum coefficient threshold and a 0.90 AIC_c_ cutoff weight

Coefficient *x* a	Max^b^_*x*_	Min^c^_*x*_	Norm^d^_*x*_	Relationship^c^
Extent				
PJ canopy cover	0.14	0.85	.50	+
Tree mound soil pH	0.21	0.56	.25	−
PJ tree ring count	0.32	0.56	.16	+
July 2009 relative humidity	0.34	0.41	.04	−
Tree mound clay content	0.36	0.40	.03	−
Severity				
PJ canopy cover	1.15	2.83	.50	+
July 2009 relative humidity	1.13	2.34	.36	−
Annual min. temperature	1.63	1.90	.08	−
Tree mound soil pH	1.68	1.75	.02	−
PJ width	1.70	1.77	.02	+
PJ trunk diameter	1.70	1.77	.02	+
Thickness				
Tree mound clay content	0.90	2.70	.48	−
PJ canopy cover	1.03	2.48	.39	+
Annual min. temperature	0.08	0.31	.06	−
Annual max. temperature	1.47	1.58	.03	−
PJ height	1.50	1.59	.02	+
Burn severity	1.51	1.56	.01	+

Norm_*x*_ values range between 0 and 1; more influential coefficients have higher Norm_*x*_ values; PJ, piñon–juniper.

a Coefficients found to be significant in the model average.

b Model average product when coefficient *x* is held at its maximum field value and all others are held at their median.

Model average product when coefficient *x* is held at its minimum field value and all others are held at their median.

Normalized influence statistic; Max_*x*_ *‐*Min_*x*_ divided by the sum of all Max_*x*_ *‐*Min_*x*_ values.

c Relationship to the soil water repellency characteristic of interest, “+” indicates a positive relationship, “−” indicates a negative relationship.

## 4 DISCUSSION

### 4.1 Soil water repellency extent and severity, and the thickness of the water repellent layer

The impact of SWR on hydrologic patterns is related to the continuity and strength of the water‐repellent layer (Doerr et al., 2009; Neris, Tejedor, Rodríguez, Fuentes, & Jiménez, 2013; Pierson et al., 2001; Woods et al., 2007). Thus, as SWR extent and severity increase, the influence of SWR on postfire recovery increases. Ten percent of our sites evidenced SWR extent above 75%, suggesting that SWR affected postfire hydrology and recovery within many of the expansion woodlands studied.

The extent of SWR varied largely among sites. This variation may be explained in part by differences in woody species composition. Microsite comparisons indicate that SWR extent and severity, and the thickness of the water‐repellent layer are greater beneath woody species—sites with higher cover would proportionally have more of these tree/shrub mound zones. Even in the interspaces, closer proximity to woody plants resulted in greater probability of SWR presence.

Comparisons between soils below singleleaf piñon and Utah juniper indicate that overstory species differences may also influence SWR. All SWR attributes were greatest under singleleaf piñon, especially severity and the thickness of the water‐repellent layer; severity (i.e., WDPT) was 96% greater and the water repellent layer was 77% thicker under singleleaf piñon compared to Utah juniper. Soil water repellency has often been related to woody species composition (Doerr et al., 2000 and references therein; Jiménez‐Pinilla, Lozano, et al., 2016). The resins, waxes, and aromatic oils contained in some woody species, particularly evergreens, are one of the primary sources of water‐repellent compounds. Indeed, water repellency develops as the litter (McGhie & Posner, 1981) and root exudates (Doerr, Shakesby, & Walsh, 1998) of these species are incorporated into the soil (Doerr et al., 2000).

These findings give weight to the argument that the effect of SWR on postfire recovery is closely tied to the prevalence, arrangement, and species of woody vegetation. Where cover of some woody species is high, SWR extent is more likely to be contiguous enough to induce overland flow and thereby accelerate site degradation postfire.

### 4.2 Ecological site characteristic modeling

Piñon–juniper canopy cover was found in all of the top models of SWR extent and many of the top models of SWR severity and the thickness of the water‐repellent layer. In every case, piñon–juniper canopy cover exhibited a positive relationship with SWR. In addition, piñon–juniper canopy cover was the most influential variable for SWR extent and severity and the second most influential variable for the thickness of the water‐repellent layer.

In the Intermountain West, the majority of piñon–juniper woodlands are only in the mid‐stages of stand closure (Miller et al., 2008). Infilling is expected to increase canopy cover over the next 30‐50 years (Miller et al., 2008; Weisburg, Lingua, & Pillai, 2007). As crown closure continues, the frequency of large‐scale, high intensity wildfires could rise (Gruell, 1999; Miller & Tausch, 2001), increasing the overall area that burns within piñon–juniper woodlands. The results of this study indicate that if this occurs, a greater proportion of burned areas will exhibit SWR.

Piñon–juniper tree ring count, tree mound soil pH, and relative humidity prior to the fire held the greatest influence on SWR next to piñon–juniper canopy cover. The relationship between SWR and piñon–juniper tree ring count is straightforward. As mentioned previously, the litter of some woody species is a primary source of water‐repellent compounds. It follows that as piñon–juniper individuals age, the quantity of water‐repellent particles in the soil would increase, improving the likelihood of SWR formation.

The relationship between SWR and tree mound soil pH may be explained in part by litter, specifically the effect of litter on pH. In some cases, when litter is incorporated into the soil, pH declines (Facelli & Pickett, 1991; Frost & Edinger, 1991). As litter is a source of water repellent particles, it is logical that water repellent areas would exhibit lower pH levels.

Relative humidity the month prior to fire was important in many of the top predictive models of SWR extent and severity. Relative humidity has been shown to directly influence SWR measurements, with SWR typically being enhanced at high relative humidity levels (Jiménez‐Pinilla, Doerr, et al., 2016; Jiménez‐Pinilla, Lozano, et al., 2016). Our research, however, demonstrates that SWR increases with decreasing relative humidity levels. This may be because relative humidity has a strong relationship on the intensity of a wildfire (Torn & Fried, 1992) and thereby may control which areas reach the temperatures necessary to generate SWR (DeBano et al., 1976; Letey, 2001). Additionally, relative humidity influences soil moisture content (Douville, Viterbo, Mahfouf, & Beljaars, 2000; Mahfouf, 1991). Soil moisture decreases the formation of SWR by moderating soil temperatures at the time of fire (Doerr & Thomas, 2000; Doerr et al., 2000, 2006; Horne & McIntosh, 2000).

Reductions in soil moisture and relative humidity brought on by climate change could intensify SWR in piñon–juniper woodlands. According to Goebel et al. (2011), climate change intensified SWR may exacerbate the effects of climate drought and detrimentally affect vegetation and microbial community structure. In combination with continuing crown closure and subsequent increasing fire frequency and intensity in these woodlands, the results of this study effectively support the claim that the effects of SWR on the recovery of piñon–juniper woodlands could intensify in the near future.

To meet this threat, this study provides simple predictive models of SWR extent and severity that allow land managers to predict SWR at the scale of their treatments without having to gather in situ data. Both endorsed models include the same two variables, piñon–juniper canopy cover and relative humidity. Piñon–juniper canopy cover can be quickly extracted over large spatial extents from remotely sensed imagery (Davies et al., 2010; Hulet et al., 2014; Madsen, Zvirzdin, Davis, Petersen, & Roundy, 2011) and relative humidity data can be easily calculated from climate datasets available in GIS format online. The parsimonious nature of these models, in combination with the remote accessibility of their coefficients, increases the likelihood that these models will be accurate when employed, although additional research is needed to validate these models.

## 5 CONCLUSIONS

Postfire SWR is widespread within piñon–juniper woodlands and is almost always found in the tree mound zones of piñon–juniper individuals or closely adjacent. Of the ecological site characteristics studied, piñon–juniper canopy cover had the strongest relationship to SWR extent and severity. Piñon–juniper tree ring count, soil pH, and relative humidity were also important. The balance of the other ecological site characteristics studied lacked strong, consistent relationships with SWR.

The strong relationship between piñon–juniper canopy cover and SWR extent leads us to conclude that where piñon–juniper canopy cover is high, SWR can be contiguous enough to induce changes in overland flow and alter hydrologic processes. As these woodlands increase in cover, a greater proportion of piñon–juniper woodlands may develop SWR during wildfires. In addition, decreases in relative humidity and soil moisture brought about by a changing climate may increase the frequency and intensity of wildfires, which could produce sites with stronger SWR over a greater spatial extent. An increase in SWR could impair natural recovery after a wildfire and result in greater challenges in postfire restoration efforts.

The results of this study suggest that piñon–juniper canopy cover and relative humidity data can be used in concert to predict SWR following fire. These data are remotely available and can be quickly derived from high‐resolution aerial photography and cloud‐based climate datasets. Using these data in conjunction with the predictive models endorsed herein, managers are provided with a means to identify potential problem areas and thereby prioritize treatment. As land managers typically have limited resources to monitor the extensive landscapes they are responsible for, this study provides an economical means for assessing a soil condition that is commonly found in the postfire piñon–juniper landscapes of the Intermountain West. As threats to natural landscapes intensify in the coming years, tools such as those provided in this study will be increasingly sought after to aid managers in making informed decisions.

## CONFLICT OF INTEREST

None declared.
